# Microarray dataset of gene transcription in mouse microglia and peripheral monocytes in contextual fear conditioning

**DOI:** 10.1016/j.dib.2022.108862

**Published:** 2022-12-27

**Authors:** Zhiqian Yu, Mai Sakai, Hotaka Fukushima, Chiaki Ono, Yoshie Kikuchi, Ryuta Koyama, Ko Matsui, Tomoyuki Furuyashiki, Satoshi Kida, Hiroaki Tomita

**Affiliations:** a Department of Psychiatry, Graduate School of Medicine, Tohoku University, Sendai, Japan; b Tohoku Medical Megabank Organization, Tohoku University, Sendai, Japan; c Department of Psychiatry Nursing, Tohoku University, Sendai, Japan; d Department of Bioscience, Faculty of Life Sciences, Tokyo University of Agriculture, Japan; e Laboratory of Chemical Pharmacology, Graduate School of Pharmaceutical Sciences, The University of Tokyo, Japan; f Super-network Brain Physiology, Tohoku University Graduate School of Life Sciences, Sendai, Japan; g Division of Pharmacology, Kobe University Graduate School of Medicine, Kobe, Japan; h Graduate School of Agriculture and Life Sciences, The University of Tokyo, Tokyo, Japan; i Department of Disaster Psychiatry, International Research Institute for Disaster Science, Tohoku University, Sendai, Japan

**Keywords:** Contextual fear conditioning, Microglia, Peripheral monocytes, Microarray

## Abstract

The transcription profile of microglia related to fear conditioning remains unclear. Here, we used Illumina MouseWG-6v2 microarrays to investigate the gene transcription changes in microglia and peripheral monocytes after contextual fear conditioning of C57BL/6 J mice. Mice were trained with or without a single minimized footshock stimulation (0-s or 2-s, 0.4 mA) and re-exposed to the training context without footshock for three different durations 24 h later: 0 min (FS0), 3 min (FS3), or 30 min (FS30). Whole brain microglia and peripheral monocytes were prepared 24 h after re-exposure using a neural tissue dissociation kit, including non-footshock controls for two re-exposure durations (Con3 and Con30). The data can be valuable for researchers interested in glial cells and neurotransmission studies and are related to the research article “Contextual fear conditioning regulates synapse-related gene transcription in mouse microglia”.

**Specifications Table**

**Table:** 

Subject	Neuroscience: Behavioral
Specific subject area	Glial cells
Type of data	Tables and figures
How the data were acquired	Illumina MouseWG-6 v2.0 Expression beadchip
Data format	Normalized data txt. zip.
Description of data collection	Male mice (C57BL/6 J; 7–8 weeks old) were exposed to contextual fear conditioning by footshock and followed with a short-duration (retention of fear memory) or long-duration (extinction of fear memory) reexposure. Whole brain microglia and peripheral monocytes were isolated from those footshocked mice, and controls (without footshock). All samples are collected and subjected to total RNA extraction, and the quality of total RNA is checked. Microarray analysis was performed by Illumina MouseWG-6v2 Expression BeadChips.
Data source location	Department of Psychiatry, Graduate School of Medicine, Tohoku University. 1–1 Seiryo-machi, Aoba-ku, Sendai, Miyagi-ken, Japan
Data accessibility	The microarray data are publicly available at the ArrayExpress database at EMBL-EBI under accession number E-MTAB-12,361. Repository name: ArrayExpress Data identification number: E-MTAB-12,361 Direct URL to data: https://www.ebi.ac.uk/biostudies/arrayexpress/studies/E-MTAB-12361?query=E-MTAB-12361
Related research article	Zhiqian Yu, Mai Sakai, Hotaka Fukushima, Chiaki Ono, Yoshie Kikuchi, Ryuta Koyama, Ko Matsui, Tomoyuki Furuyashiki, Satoshi Kida, Hiroaki Tomita. Contextual fear conditioning regulates synapse-related gene transcription in mouse microglia. *Brain Res Bull*. 2022 Oct 15;189:57–68. https://doi.org/10.1016/j.brainresbull.2022.08.017. [1]

## Value of the Data

• The data help understand the gene transcription changes in microglia and peripheral monocytes in contextual fear conditioning.
• The data help understand the gene transcription changes in microglia and peripheral monocytes in contextual fear conditioning.
• The data can be used to identify target pathways/transcriptions to improve the relationship between microglia and neurotransmission.
• The data could be used by investigators interested in microglial and peripheral transcriptions and molecular mechanisms in psychiatric disorders associated with fear memory, such as post-traumatic stress disorders.

## 1 Objective

The original published article did not provide the datasets of microarray. The microarray datasets will help to understand more information about the original research and provide further analysis to understand the gene transcription changes in the microglia and peripheral monocytes in contextual fear conditioning.

## 2 Data Description

**Fig. 1:**
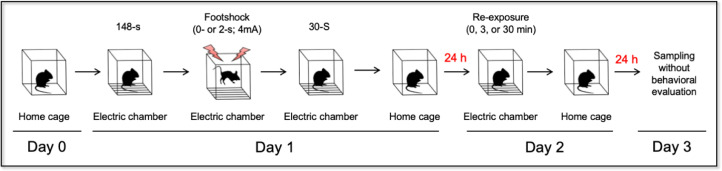
Schedule of the five contextual conditions. Control mice were trained without receiving footshocks (0-s, 4 mA) and re-exposed to the context for 3 min (Con3) or 30 min (Con30). Both footshocked mice (2-s, 4 mA) with 3 min (FS0) or without re-exposure (FS0) result in retention of fear memory, whereas 30 min re-exposure (FS30) facilitates extinction of fear memory. Sampling, whole brain microglia and peripheral monocytes.

**Fig. 2:**
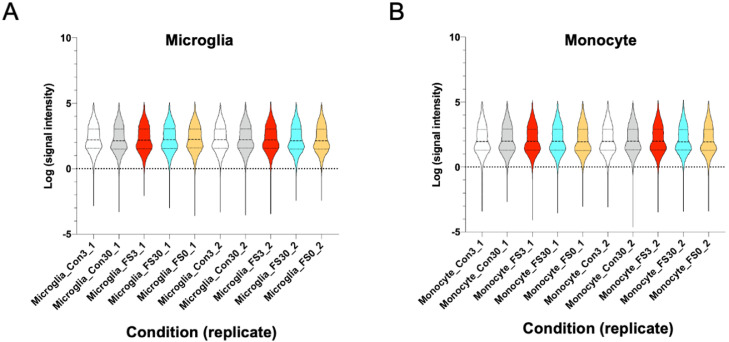
Violin plots of signal intensity. (A) After average normalization, the log 10 transformed signal intensity of each microglial sample was shown as violin plots with median (dotted line), inter-quartile range (solid line). (B) After average normalization, the log 10 transformed signal intensity of each monocytic sample was shown as violin plots with median (dotted line), inter-quartile range (solid line).

RNA was extracted from whole-brain microglia and peripheral monocytes from C57BL/6 J mice in five conditions (Fig. 1) with two biological replicates (Fig. 2). In detail, the RNA samples of isolated whole brain microglia (*n* = 20) in each of five conditions (Con3, Con30, FS0, FS3, and FS30) were randomly divided into two groups (*n* = 10) and pooled for one microarray (*n* = 2; 5 conditions each). Similarly, the RNA samples of isolated peripheral monocytes (*n* = 20) in each of five conditions (Con3, Con30, FS0, FS3, and FS30) were randomly divided into two groups (*n* = 10) and pooled for one microarray (*n* = 2; 5 conditions each).

The normalized signal intensity and text data of Illumina MouseWG-6 v2.0 Expression beadchip are provided as supplementary files (FinalReport_Microglia.txt and FinalReport_Monocyte.txt) at ArrayExpress database (https://www.ebi.ac.uk/arrayexpress) with accession number E-MTAB-12,361. The link between the microarray ID and the microarray data are provided in Table 1, and the supplementary file (SampleID.txt) at ArrayExpress database (E-MTAB-12,361). The distributions of normalized signal intensities are shown in Fig. 2. Information on Illumina ID are provided as supplementary file (IlluminaID.txt) at the ArrayExpress database (E-MTAB-12,361).

**Table 1:** RNA sample and cell types associated with microarray ID (*n* = 2; 5 conditions each).

Microarray ID	Cell Type	Conditions (replicate)
9,257,615,127_A:AVG_Signal	Microglia	Con3_1
9,257,615,127_B:AVG_Signal	Microglia	Con30_1
9,257,615,127_C:AVG_Signal	Microglia	FS3_1
9,257,615,127_D:AVG_Signal	Microglia	FS30_1
9,257,615,127_E:AVG_Signal	Microglia	FS0_1
9,257,615,133_A:AVG_Signal	Microglia	Con3_2
9,257,615,133_B:AVG_Signal	Microglia	Con30_2
9,257,615,133_C:AVG_Signal	Microglia	FS3_2
9,257,615,133_D:AVG_Signal	Microglia	FS30_2
9,257,615,133_E:AVG_Signal	Microglia	FS0_2
9,249,051,032_A:AVG_Signal	Monocyte	Con3_1
9,249,051,032_B:AVG_Signal	Monocyte	Con30_1
9,249,051,032_C:AVG_Signal	Monocyte	FS3_1
9,249,051,032_D:AVG_Signal	Monocyte	FS30_1
9,249,051,032_E:AVG_Signal	Monocyte	FS0_1
9,249,051,050_A:AVG_Signal	Monocyte	Con3_2
9,249,051,050_B:AVG_Signal	Monocyte	Con30_2
9,249,051,050_C:AVG_Signal	Monocyte	FS3_2
9,249,051,050_D:AVG_Signal	Monocyte	FS30_2
9,249,051,050_E:AVG_Signal	Monocyte	FS0_2

Supplementary Table 1 provides the result of differential gene transcription of microglia after fear memory consolidation and extinction. In detail, the mean value of microarray data in each condition was compared separately. After fear memory consolidation, 2546 probes were increased, and 2286 probes were decreased in microglia (FS0/Con3). After fear memory extinction, 2400 probes increased, and 3074 probes decreased in microglia (FS30/FS0), which is done by increased fold change > 1.2 and decreased fold change 〈 0.833 with signal intensity 〉 50.

## 3 Experimental Design, Materials and Methods

### 3.1 Mice

C57BL/6 J male mice (7–8 weeks old) were obtained from SLC Inc. (Hamamatsu, Japan). Mice were maintained on a standard 12:12 h light/dark cycle with ad libitum access to food and water during the experimental phase. All mice were habituated by handling for 5 min/day for 5 consecutive days before experiments.

### 3.2 Contextual fear conditioning

Mice were divided into five groups each containing 20 mice per group. Mice were placed into the training chamber (17.5 × 17.5 × 15 cm) with a stainless-steel rod floor used to deliver footshocks (Ohara & Co. Ltd., Tokyo, Japan, year 2015). In Fig. 1, each mouse was transferred from its home cage to the training chamber (2:00PM - 4:00PM) and allowed to explore it for 148 s before receiving a single 2-s footshock (0.4 mA) and consecutive 30-s exposures. Twenty-four hours after conditioning, mice were re-exposed to the training chamber without receiving footshocks for varying durations as follows: 0 min (FS0; footshocked mice without re-exposure), 3 min (FS3; footshocked mice with 3 min of re-exposure), or 30 min (FS30; footshocked mice with 30 min of re-exposure) and then transferred to their home cages (Fig. 1). Both FS0 and FS3 result in retention of fear memory, whereas FS30 facilitates extinction of fear memory [1,2]. Control mice were trained without receiving footshocks and re-exposed to the context for 3 min (Con3) or 30 min (Con30) (Fig. 1). Mice were sacrificed 24 h after the re-exposure without behavioral evaluation. This procedure was performed instead of behavioral monitoring to avoid the effect of tertiary exposure to the training chamber on the molecular phenotype of the microglial cells and peripheral monocytes.

### 3.3 Isolation of microglia and peripheral monocytes

Mice were sacrificed by decapitation, and brains were prepared as a single-cell suspension using a neural tissue dissociation kit (130–093–231; Miltenyi Biotec, Bergisch Gladbach, Germany) by gentleMACS Dissociator (130–093–235; Miltenyi Biotec). CD11b-positive microglia were isolated using CD11b-labeled MicroBeads (130–093–634; Miltenyi Biotec) and autoMACS Pro-SeparatorS (130–092–545; Miltenyi Biotec). The purity of CD11b+ cells (> 98%) was confirmed using a FACSCalibur Flow Cytometer Resource (RRID: SCR_000879) (BD Bioscience, Franklin Lakes, NJ, USA) with PE anti-human and -mouse CD11b (RRID:AB_2,654,644) (130–109–363; Miltenyi Biotec), and stained using rat anti-mouse CD11b FITC-conjugated monoclonal antibodies (130–110–610; Miltenyi Biotec) for 5 min at 4  °C. After washing, the cells were subjected to flow cytometry using an ACCURI Flow Cytometer (RRID: SCR_014422) (Accuri Cytometers, Inc., Ann Arbor, MI, USA). A total of 1.81 − 2.14  ×  10^6^ microglia per mouse were collected, and the purity of CD11b-positive cells was > 97%.

The peripheral blood of mice was collected with a lithium-heparin blood collection tube (BD:366,667) (BD Vacutainer™, Franklin Lakes, NJ, USA), and mononuclear cells were separated from erythrocytes and leukocytes using Lympholyte M (Cedarlane Laboratories, Hornby, Ontario, CANADA). Monocytes were isolated using CD11b (Mac-1α) MicroBeads (130–049–601; Miltenyi Biotec) with the autoMACS Pro-SeparatorS. The purity of CD11b-positive cells was > 98% by using the ACCURI Flow Cytometer.

### 3.4 RNA extraction

Total RNA was extracted from whole brain microglia and peripheral monocytes from the contextual fear conditioning (FS0, FS3, and FS30) and those controls groups (Con3 and Con30) using the RNeasy Mini Kit and the RNeasy MinElute Cleanup Kit (74,104; Qiagen, Valencia, CA, USA). The RNA integrity number (RIN) for each RNA sample was confirmed to be > 9.8 using the Agilent 2100 Bioanalyzer (Agilent, Santa Clara, CA, USA), and the ribosomal RNA 28S/18S ratio was >1.9.

### 3.5 Microarray

Biotinylated cRNA was synthesized and applied to Illumina BeadChips from the Con3, Con30, FS0, FS3, and FS30 groups according to the manufacturer's instructions. Biotinylated cRNA was prepared from 500 ng of total RNA using the Illumina Ambion RNA Amplification Kit (Ambion, Austin, TX, USA). The biotinylated cRNA samples were hybridized to Illumina MouseWG-6v2 Expression BeadChips (Illumina, San Diego, CA, USA). Each BeadChip was washed and scanned using an Illumina Bead Station 500X. Signal intensities of each BeadChip were normalized by using "average" normalization (Fig. 2).

## Supplementary Materials

Supplementary material associated with this article can be found in the online version at: https://data.mendeley.com/public-files/datasets/jjfzr6xhb2/files/b76b08e8-deab-4549-9c8d-932164f9457c/file_downloaded.

## Ethics Statements

All experiments were conducted in accordance with the National Institutes of Health Guidelines for the Care and Use of Experimental Animals (NIH Publication No. 8023, revised 1978) and approved by the Guidelines for the Care of Laboratory Animals of Tohoku University Graduate School of Medicine (Sendai, Japan) (2018IRIDeS-001–02), 2), which mice were exposed to minimize footshock stimulation.

## CRediT Author Statement

**Zhiqian Yu, Hotaka Fukushima, Satoshi Kida** and **Hiroaki Tomita:** Designed the experiments; **Zhiqian Yu, Mai Sakai, Chiaki Ono** and **Yoshie Kikuchi:** Performed the animal experiments and behavioral tests and prepared the mouse RNA samples; **Zhiqian Yu, Mai Sakai, Ko Matsui** and **Tomoyuki Furuyashiki:** Performed the microarray analyses.

## Declaration of Competing Interest

The authors declare that they have no known competing financial interests or personal relationships that could have appeared to influence the work reported in this paper.

## Data Availability

Supplementary Table 1 (Original data) (Mendeley Data).
